# Progress against lung cancer, Denmark, 2008–2022

**DOI:** 10.2340/1651-226X.2024.26180

**Published:** 2024-05-14

**Authors:** Marianne Steding-Jessen, Henriette Engberg, Erik Jakobsen, Torben Riis Rasmussen, Henrik Møller

**Affiliations:** a The Danish Clinical Quality Program and Clinical Registries (RKKP), Aarhus, Denmark; b Department of Thoracic and Vascular Surgery, Odense University Hospital, Odense, Denmark; c The Danish Lung Cancer Registry (DLCR), Odense University Hospital, Odense, Denmark; d Department of Respiratory Diseases, Aarhus University Hospital, Aarhus, Denmark; e Danish Center for Health Services Research, Aalborg University, Aalborg, Denmark

**Keywords:** Stage, incidence, survival, mortality

## Abstract

**Background and purpose:**

There has been marked progress against lung cancer in Denmark. To gain further insight into the different aspects of the improvement, we examined the stage-specific incidence rates, stage-specific survival and mortality rates.

**Materials and methods:**

We used information from the Danish Lung Cancer Registry on date of diagnosis and clinical stage to calculate age-standardised incidence rates and patient survival by sex, period and stage. Information about age-standardised lung cancer-specific mortality rates by sex and period was extracted from The Danish Health Data Authority.

**Results:**

Firstly, the decrease in incidence rates was due to a reduction in the rates of advanced stages. Secondly, there was a gradual increase in survival across all stages, and thirdly, the mortality rates gradually decreased over time.

**Interpretation:**

The improvements in survival and mortality from lung cancer were due to decreasing incidence rates of advanced cancer and improvement in survival at all stages of the disease.

## Background

During the past decade, age-standardised lung cancer incidence has decreased in the Danish population, and the survival of lung cancer patients has improved markedly. These improvements for lung cancer in Denmark contribute to a decrease in the rate of lung cancer deaths in the population [1]. To gain further insight into the different aspects of the improvement, we examine the stage-specific incidence rates, stage-specific survival and mortality rates from lung cancer in Denmark, 2008–2022.

## Materials and methods

The Danish Lunger Cancer Register (DLCR) is based on extracts from the National Patient Register (NPR) on incident patients registered with International Classification of Diseases 10th Revision (ICD10) codes C33 and C34, which are further validated by clinicians [2]. From DLCR, we extracted information about the date of diagnosis and clinical stage according to the 8th version of the Tumor, Node, Metastasis (TNM) classification for 71,555 lung cancer patients (WHO ICD10: C33 and C34) diagnosed from 2008 to 2022 [2]. Information about the clinical stage was missing in 12% of the cases in the early period (2008–2012), in 5% in the middle period (2013–2017) and in 2% in the latest period (2018–2022). To account for missing information about the clinical stage, we used the SAS procedure IM to impute the clinical stage where it was missing based on period, age, sex and time at risk and death [3].

### Incidence

We calculated age-standardised incidence rates stratified by sex and clinical stage (IA, IB, II, III, and IV) during three time periods from 2008–2012, 2013–2017, and 2018–2022, using the Danish population on January 1, 2022, as the standard population.

### Survival

We estimated patient survival using the period analysis approach to provide up-to-date estimates of 5-year survival functions [4, 5]. Patients were followed up for death, migration, or end of follow-up on June 10, 2023.

### Mortality

Information about age-standardised lung cancer-specific mortality rates by sex and period was extracted from The Danish Health Data Authority [6]. The standard population was the Danish population on January 1, 2000.

## Results

### Incidence

**Figure 1:**
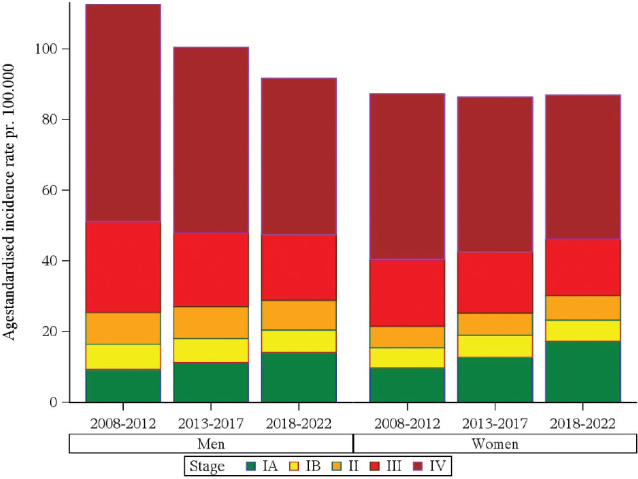
Age-standardized incidence rates for 71,555 Danish lung cancer patients, 2008–2022.

Figure 1 shows the age-standardised incidence rates stratified by sex, time period, and clinical stage. Overall, the incidence rate decreased for men and remained stable for women during the period. For both men and women, the incidence rates of very early-stage disease (stage IA) increased over time, whereas the rates of stage IV decreased. The decrease in stage IV was most pronounced for men with a decrease of 17.2 pr. 100,000 over the study period, whereas for women the decrease was 6.2 pr. 100,000. The increase in incidence of stage IA was higher for women than for men with increases of 7.5 pr. 100,000 for women and 4.8 pr. 100,000 for men. The decrease in incidence among men was mainly due to the markedly decrease in stage IV cancers, whereas for women there has been a shift toward more early stages.

### Survival

**Figure 2:**
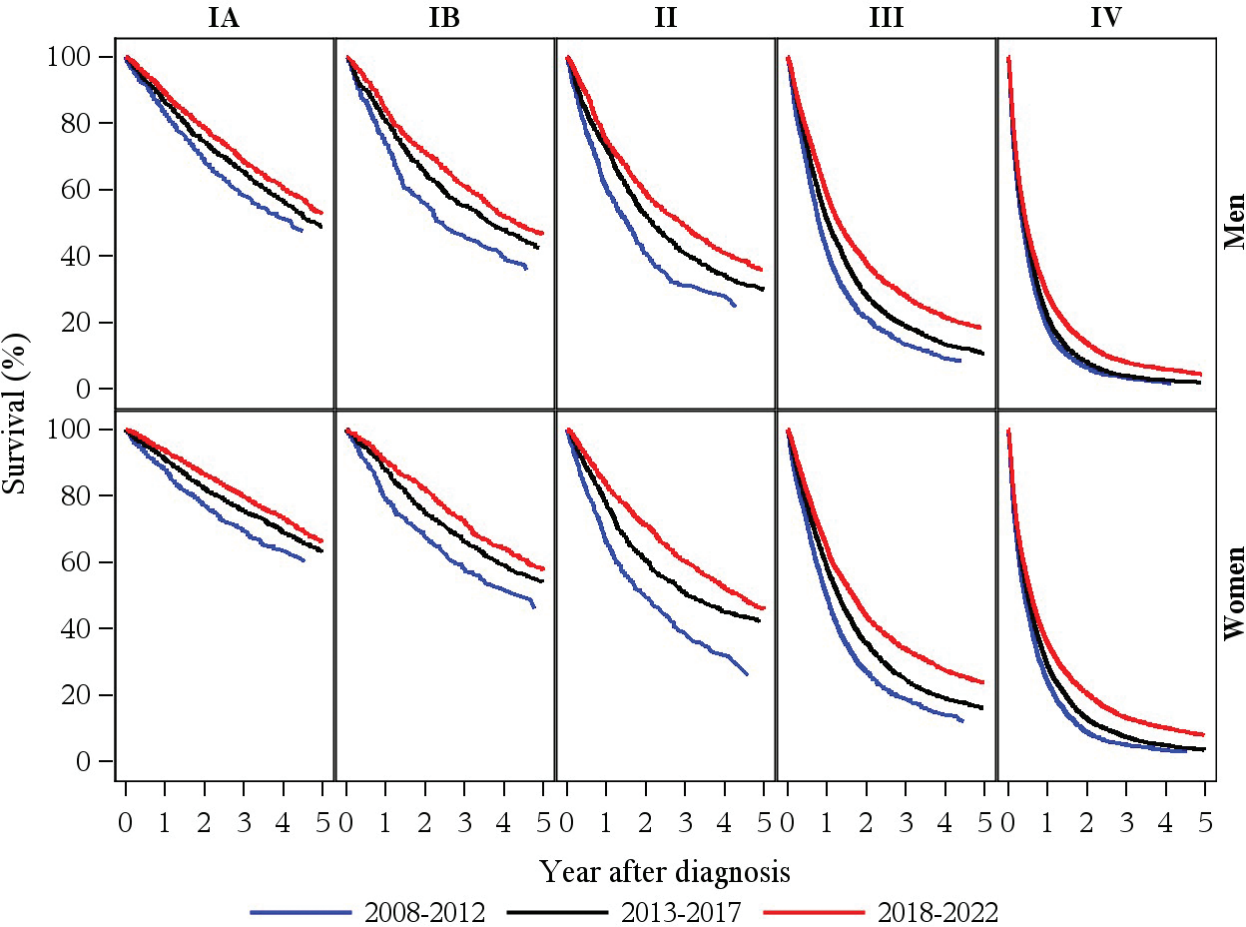
Kaplan-Meier survival functions for 71,555 Danish lung cancer patients, 2008–2022.

Figure 2 shows the Kaplan–Meier survival functions stratified by sex, clinical stage, and period. For both men and women, and across all stages, the pattern of a gradual increase in survival over time was evident. Survival improved the most for stage II and III patients with increases in 5-year survival estimates of 4–8% from the middle period, 2013–2017 to the latest period, 2018–2022. For the advanced stage IV, the survival improvement was most pronounced in the latest period, 2018–2022 with increases in 5-year survival estimates from 2% to 5%.

### Mortality

**Figure 3:**
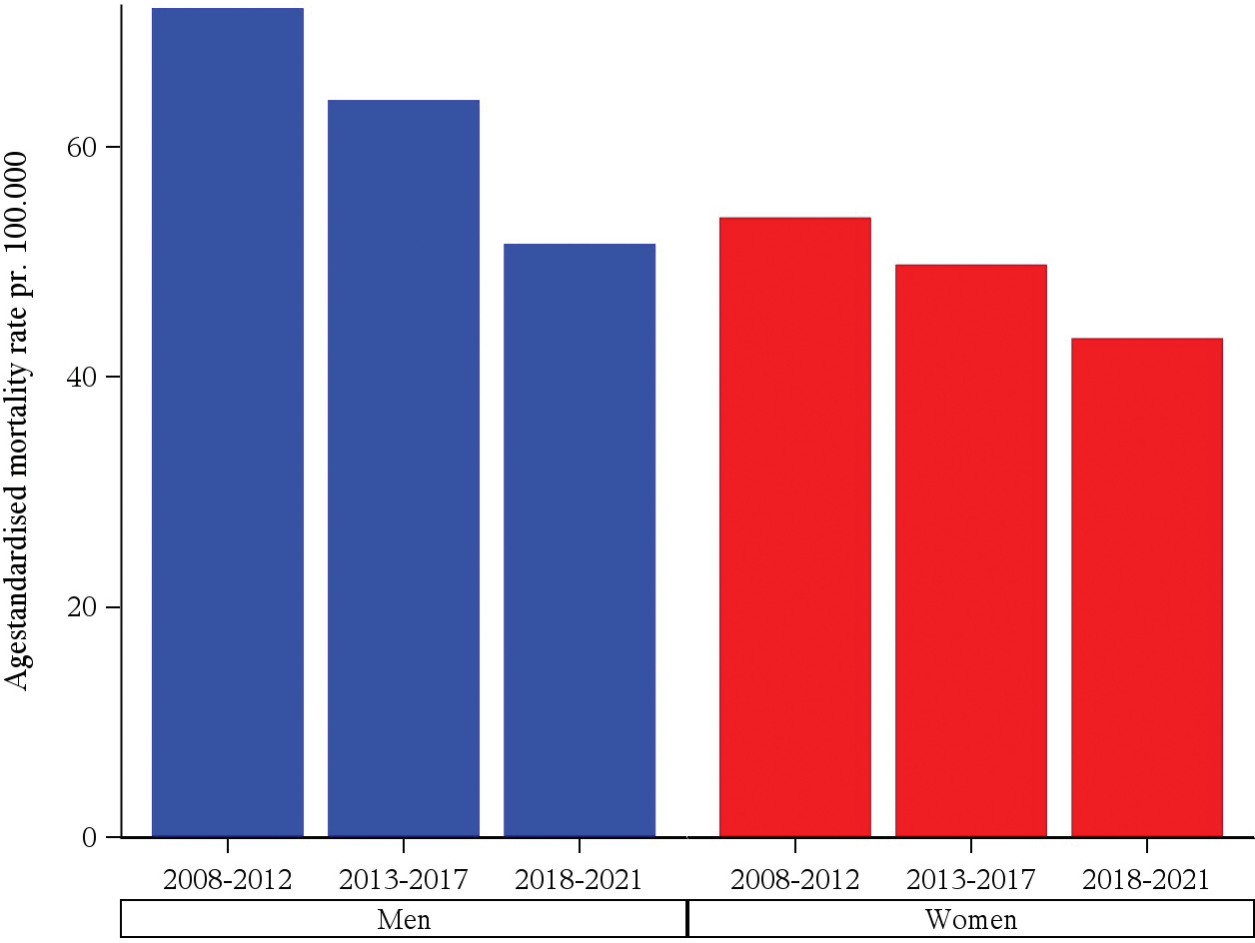
Lung cancer age-standardized mortality rates, Denmark 2008–2021.

Figure 3 shows age-standardised mortality rates by sex and time period. The mortality rates gradually decreased over time for both men and women. The decrease was most pronounced in men with a decrease from 72 pr. 100,000 to 52 pr. 100,000. The decrease for women was half of the decrease that was seen for men, from 54 pr. 100,000 to 43 pr. 100,000.

## Discussion

The long-term downward trend in age-standardised lung cancer incidence was due to reduction in the advanced-stage lung cancer for men while for women incidence rates were largely unchanged from 2008 to 2022. Moreover, there was an increase in the incidence of early-stage cancers for both men and women.

Preventive strategies such as campaigns against smoking have likely had a favorable impact on incidence and mortality from lung cancer. The long-term downward trend in incidence of advanced lung cancer may be due to a reduction in the prevalence of tobacco smoking over successive generations together with an increased awareness of the symptoms of lung cancer leading to an earlier examination and thus diagnosis at an earlier stage. But smoking and smoking cessation together with an increased awareness have impacted incidence rates differently in men and women. The male population adopted the smoking habit earlier and to a greater extent than women, and there has been an earlier and more pronounced decline in the prevalence of smoking in men. The incidence in women peaks at a later time point than in men.

The improved survival for stage III and IV patients may be due to improved treatment such as personalized medicine and immunotherapy, which has become increasingly available during the study period. Furthermore, these new treatments have been particularly beneficial for patients with adenocarcinoma, of which there has been an increasing proportion over the years [7]. Because stage IV lung cancer constitutes nearly half of all lung cancer cases, the improvement in stage IV survival has had at major impact on overall lung cancer survival and mortality. The decline in mortality expresses contributions from declining incidence, from a higher proportion of patients diagnosed with lung cancer in curable stages and from prolonged survival in patients with advanced cancer.

A possible explanation for the increase in stage IA lung cancer incidence may be an increase in the use of diagnostic CT imaging in Denmark during the period. Such imaging may be rational and useful with respect to the primary indication for its use, but it entails an opportunity of detection of lung nodules representing early-stage lung cancer. Eighty-five per cent of stage IA lung cancers in Denmark are currently found as incidental findings on computer tomography (CT) imaging for other reasons than suspicion of lung cancer [8]. Some of these incidentally detected lung cancers may be slow-growing cancers that would not have caused symptoms and illness if not detected incidentally [9]. The improvement in survival for stage I and stage II lung cancer patients may be due to better diagnostic tools and imaging procedures to secure correct staging and thereby optimal treatment. The possible overdiagnosis of indolent stage IA cancers enriches the patient population with good-prognosis cases and may hereby contribute to increasing survival estimates.

It has been decided to pilot a screening program for lung cancer in heavy smokers and ex-smokers in Denmark, as screening based on low-dose CT has the demonstrated potential to reduce the population mortality rate from lung cancer [10]. It is important that the pilot makes attempt to estimate the extent of overdiagnosis and the added workload associated with the monitoring of suspect noduled found by screening.

In conclusion, there has been a major improvement in the survival of lung cancer patients and in population mortality from lung cancer in Denmark. The main drivers are the decreasing incidence rates of advanced cancer and improvement in the survival of lung cancer patients at all stages of the disease. A large increase in incidence of stage IA lung cancer was most likely due to increased use of CT imaging for a wide range of indications.

## Data Availability

Extract from the Danish Lung Cancer Register can be applied for from www.rkkp-forskning.dk, and data on mortality can be accessed from The Danish Health Data Authority, https://www.esundhed.dk/Emner/Hvad-doer-vi-af/Doedsaarsager. Accessed July 10, 2023.
